# The co-chaperone and reductase ERdj5 facilitates rod opsin biogenesis and quality control

**DOI:** 10.1093/hmg/ddu385

**Published:** 2014-07-23

**Authors:** Dimitra Athanasiou, Dalila Bevilacqua, Monica Aguila, Caroline McCulley, Naheed Kanuga, Takao Iwawaki, J. Paul Chapple, Michael E. Cheetham

**Affiliations:** 1 UCL Institute of Ophthalmology, London EC1V 9EL, UK,; 2 Advanced Scientific Research Leaders Development Unit, Gunma University, Maebashi, Gunma371-8511, Japan and; 3 Centre for Endocrinology, William Harvey Research Institute, Barts and the London School of Medicine and Dentistry, Queen Mary University of London, London EC1M 6BQ, UK

## Abstract

Mutations in rhodopsin, the light-sensitive protein of rod cells, are the most common cause of autosomal dominant retinitis pigmentosa (ADRP). Many rod opsin mutations, such as P23H, lead to misfolding of rod opsin with detrimental effects on photoreceptor function and viability. Misfolded P23H rod opsin and other mutations in the intradiscal domain are characterized by the formation of an incorrect disulphide bond between C185 and C187, as opposed to the correct and highly conserved C110–C187 disulphide bond. Therefore, we tested the hypothesis that incorrect disulphide bond formation might be a factor that affects the biogenesis of rod opsin by studying wild-type (WT) or P23H rod opsin in combination with amino acid substitutions that prevent the formation of incorrect disulphide bonds involving C185. These mutants had altered traffic dynamics, suggesting a requirement for regulation of disulphide bond formation/reduction during rod opsin biogenesis. Here, we show that the BiP co-chaperone and reductase protein ERdj5 (DNAJC10) regulates this process. ERdj5 overexpression promoted the degradation, improved the endoplasmic reticulum mobility and prevented the aggregation of P23H rod opsin. ERdj5 reduction by shRNA delayed rod opsin degradation and promoted aggregation. The reductase and co-chaperone activity of ERdj5 were both required for these effects on P23H rod opsin. Furthermore, mutations in these functional domains acted as dominant negatives that affected WT rod opsin biogenesis. Collectively, these data identify ERdj5 as a member of the proteostasis network that regulates rod opsin biogenesis and supports a role for disulphide bond formation/reduction in rod opsin biogenesis and disease.

## INTRODUCTION

Rhodopsin, the light-absorbing photopigment of rod cells, is the archetypal G-protein-coupled receptor (GPCR) and is composed of the apoprotein rod opsin and the covalently linked 11-*cis* retinal chromophore, an analogue of vitamin A. The apoprotein rod opsin is synthesized in the rough endoplasmic reticulum (ER) membrane in the inner segments of rod photoreceptor cells before being trafficked to the rod outer segment photosensory cilia. Rhodopsin undergoes several post-translation modifications during its biogenesis, including the formation of a disulphide bond between C110 and C187 residues in the intradiscal domain (1–4). This disulphide bond is highly conserved among other GPCRs such as the β_2_ adrenergic and M_1_ muscarinic receptors (5,6).

Mutations in rod opsin cause the neurodegenerative disease retinitis pigmentosa (RP) that leads to blindness as a result of photoreceptor cell death. The first mutation associated with RP, a proline-to-histidine substitution in codon 23 (P23H), was reported in 1990 and found to cause autosomal dominant RP [autosomal dominant retinitis pigmentosa (ADRP)] (7). Since then, over 200 rhodopsin point mutations have been described, which account for ∼25% of all ADRP cases (RetNet: http://www.sph.uth.tmc/edu/Retnet/). The P23H mutation, however, is the most common mutation in North America and the most studied. The P23 residue is found in the intradiscal domain of rhodopsin. Mutations in this domain have been found to cause partial (e.g. P23H and D190A) or total misfolding (e.g. G188R, C110F, C110Y and C187Y), with limited ability to bind 11-*cis* retinal and form a functional, stable photopigment (8,9) and have been classified as Class II mutations (10). Studies of ADRP-associated mutations, as well as designed mutations in the intradiscal domain, have shown the presence of a non-native disulphide bond between C185 and C187 residues (3,8,11–13). While initially this incorrect disulphide bond was thought to be the cause of misfolding in these mutants, later it was suggested that it traps misfolded forms of rod opsin and is not the sole or direct cause of rod opsin misfolding (3).

Rod opsin folding-defective polypeptides are not allowed to traffic through the secretory system, for example, heterologous expression of P23H rod opsin showed that it is retained in the ER (14,15). ER-retained P23H rod opsin is subject to retrotranslocation and ER-associated degradation (ERAD), a process that requires the reduction of any interchain or intrachain disulphide bonds (16). Upon failure of ERAD, P23H rod opsin can spontaneously aggregate and form microscopically visible inclusion bodies (17,18). In the photoreceptors of homozygous P23H knock-in mice, the vast majority of P23H rod opsin is degraded; however, a small amount escapes the ER and traffics to ciliary protrusions where it forms elongated discs (19,20). Rod opsin misfolding can stimulate the unfolded protein response (21), which is a coordinated stress response that reduces translation, induces expression of molecular chaperones and stimulates ERAD to recover from protein misfolding stress in the ER. Previous studies have shown that molecular chaperones are important for rod opsin biogenesis and photoreceptor function (22,23), whereas pharmacological manipulation of their activity can alleviate the toxic effects of misfolded and mutant rod opsin (24–26).

Some of the factors that facilitate normal rod opsin folding and the quality control of mutant rod opsin have been discovered. The ER degradation-enhancing α-mannosidase-like 1 (EDEM1) and valosin-containing protein (VCP/p97) have been found to facilitate the degradation of P23H rod opsin (27,28). Calnexin (Cnx) overexpression can improve the folding of P23H rod opsin in the presence of 11-*cis* retinal (29). However, loss of its activity revealed no requirement for Cnx in P23H rod opsin ERAD or the biogenesis of normal rod opsin, unlike *Drosophila* rhodopsin *Rh1* (30–32). Additionally, the ER Hsp70, BiP (HSPA5), was shown to be important in preventing rod opsin aggregation in the ER, suggesting that BiP is a critical factor in the biogenesis and quality control of normal and mutant rod opsin (33) and its overexpression improves photoreceptor survival in animal models of P23H RP (34). The requirement of the ATPase activity of BiP for rod opsin biogenesis (33) suggested that an ER DnaJ protein might also be involved in rod opsin biogenesis.

ERdj5 (also known as DNAJC10 or J-containing PDI-like protein—JPDI) is the fifth member of the ERdj family of ER proteins. It has a unique structure owing to the combination of a J domain, which regulates the interaction with BiP, six thioredoxin-like (Trx) domains (four of which are active reductases and contain CXXC motifs) and a KDEL tetrapeptide, which mediates ER retention, within the same structure (35–38). ERdj5 has been found to be part of two distinct ERAD pathways that exist for the glycosylated and non-glycosylated misfolded proteins. This is due to its ability to act as a reductase that catalyses the reduction of disulphide bonds of proteins that are targeted for degradation, to act as a co-chaperone for BiP and to bind to EDEM1 (35–40). Apart from an ERAD component, ERdj5 has been recently shown to be required for the folding and secretion of the low-density lipoprotein receptor (LDLR) highlighting the diversity of its biological functions (41).

Taking into consideration the relationship of ERdj5 with BiP and EDEM1, which are both important for rod opsin biogenesis and the potential requirement for reduction of the non-native C185–C187 disulphide bond upon rod opsin misfolding, we investigated the role of ERdj5 in rod opsin biogenesis. Here, we show that correct disulphide bond formation/reduction is an important step in rod opsin biogenesis and quality control that is regulated by ERdj5. Importantly, we identify ERdj5 as member of rod opsin proteostasis network that promotes the degradation and prevents the aggregation of P23H rod opsin while facilitating the normal processing of wild-type (WT) rod opsin.

## RESULTS

### Disulphide bond formation/reduction regulates rod opsin biogenesis

To investigate the hypothesis that a non-native C185–C187 disulphide bond might affect rod opsin biogenesis, the C185 residue was substituted to alanine (C185A) or serine (C185S) on WT (Rho-GFP) and P23H (Rho(P23H)-GFP) rod opsin constructs tagged at the C-terminus with GFP (17). These substitutions were made in order to eliminate the possibility of the C185–C187 disulphide bond formation and investigate this effect on WT rod opsin, as well as in combination with the P23H mutation. The C185S, C185A and P23H-C185A mutants have been studied *in vitro* for their ability to bind to 11-*cis* retinal (1,3); however, their traffic, mobility in the ER and the combination of P23H with C185S substitution have not been described before.

**Figure 1.**
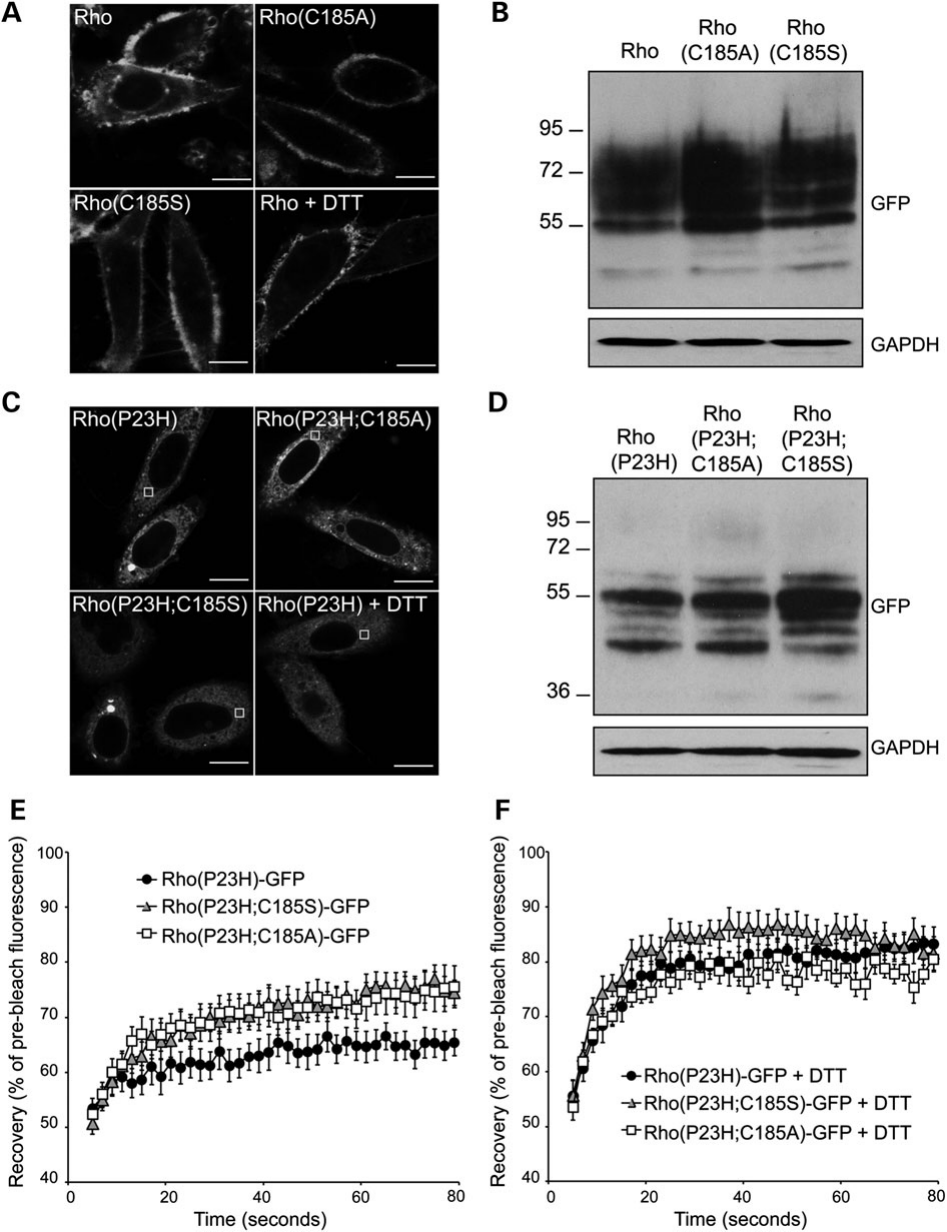
Prevention of the incorrect C185–C187 disulphide bond affects P23H rod opsin dynamics. SK-N-SH cells were transfected with either (**A, B**) Rho-GFP, Rho(C185A)-GFP and Rho(C185S)-GFP or (**C–F**) Rho(P23H)-GFP, Rho(P23H;C185A)-GFP and Rho(P23H;C185S)-GFP. (A) Representative live-cell images of Rho-GFP, Rho(C185A)-GFP, Rho(C185S)-GFP and Rho-GFP incubated for 15 min with 3 mm DTT 24 h after transfection. Scale bar: 10 μm. (B, D) Western blot of cell lysates showing Rho-GFP (B) and Rho(P23H)-GFP (D) probed with an antibody against GFP. The position of the molecular weight markers, in kDa, is shown on the left. GAPDH was used as a loading control. (C) Representative live-cell images of Rho(P23H)-GFP, Rho(P23H;C185A)-GFP and Rho(P23H;C185S)-GFP and Rho(P23H)-GFP incubated for 15 min with 3 mm DTT 24 h after transfection. Scale bar: 10 μm. (E) Graphical representation of fluorescence recovery after photobleach for Rho(P23H)-GFP (black circles), Rho(P23H;C185S)-GFP (grey triangles) and Rho(P23H;C185A)-GFP (white squares). Fluorescence intensities for the 2 × 2-μm area were normalized to pre-bleach levels at 100%. Error bars represent standard error, *n* ≥ 12. (F) Graphical representation of recovery after photobleach for Rho(P23H)-GFP (black circles), Rho(P23H;C185S)-GFP (grey triangles) and Rho(P23H;C185A)-GFP (white squares) in the presence of DTT, as indicated. Fluorescence intensities for the 2 × 2-μm area were normalized to pre-bleach levels at 100%. Error bars represent standard error, *n* ≥ 12.

SK-N-SH cells were transfected with Rho-GFP, Rho(C185A)-GFP, Rho(C185S)-GFP, Rho(P23H)-GFP, Rho(P23H;C185A)-GFP or Rho(P23H;C185S)-GFP (Fig. 1). In order to determine empirically the different rod opsin species, cell lysates were digested with the enzymes endoglycosidase H (EndoH) and peptide N-glycosidase (PNGase F) before blotting and probing with a GFP antibody. All WT rod opsin constructs migrated as a major smear of polypeptides between 55 and 90 kDa and showed the same resistance on EndoH digestion and sensitivity to PNGase F digestion (Fig. 1B; Supplementary Material, Fig. S1A). This agrees with previous reports on indistinguishable glycosylation and expression pattern between WT and C185A and C185S mutants (1,3).

Additionally, all Rho(P23H)-GFP proteins migrated as three distinctive polypeptides ranging from 40 to 70 kDa. Rho(P23H)-GFP expressed at slightly lower levels compared with Rho(P23H;C185A)-GFP and Rho(P23H;C185S)-GFP (Fig. 1D). The ∼55 kDa polypeptide corresponded to ER-resident rod opsin as it was sensitive to EndoH digestion and was enhanced in the double mutants (Supplementary Material, Fig. S1B). Incubation of all the mutants with both PNGase F and EndoH enhanced the formation of high-molecular-weight species on the top of the gel. Previously, it has been shown that the addition of GFP-tag in rod opsin increases the rate of misfolding and inclusion formation compared with untagged rod opsin (17,42).

Live imaging was performed in order to compare the ER diffusional mobility of these constructs by fluorescence recovery after photobleaching (FRAP) as previously described (33) (Fig. 1A and C); however, it was not possible to perform FRAP in Rho(C185A)-GFP- and Rho(C185S)-GFP-expressing cells because they trafficked more efficiently to the plasma membrane, compared with Rho-GFP, and there was no discernible GFP fluorescence in the ER (Fig. 1A). The same effect was observed with Rho-GFP after 15-min incubation with the reducing agent dithiothreitol (DTT), which reduces disulphide bonds and inhibits their formation (43,44) (Fig. 1A). Overall these results suggest that C185A and C185S substitutions do not have a detrimental effect on the folding and maturation of Rho-GFP in the ER. In contrast, prevention of the non-native disulphide bond is likely to lead to a faster trafficking of Rho-GFP to the plasma membrane, suggesting that formation of an incorrect disulphide bond might limit the folding rate for WT rod opsin.

Live imaging of the mutant proteins showed that similar to Rho(P23H)-GFP, both double mutants were retained in the ER and no clear plasma membrane localization was observed (Fig. 1C). FRAP was performed in low-expressing, large-inclusion-free cells and showed that the recovery of Rho(P23H)-GFP was ∼65% of pre-bleached levels, similar to previous studies of Rho(P23H)-GFP mobility (33) (Fig. 1E). In contrast, both double mutants were more mobile and showed similar levels of recovery that reached ∼75% of pre-bleached levels (Fig. 1E). This suggested that both double mutants were more soluble in the ER possibly as a result of the prevention of abnormal disulphide bond formation.

Within 15-min incubation of the cells with 3 mm DTT, Rho(P23H)-GFP showed 20% increase in its recovery, which reached ∼85% recovery of the pre-bleached levels. Interestingly, treatment with DTT led to a 4% increase in the recovery of Rho(P23H;C185A)-GFP and a 7% increase in Rho(P23H;C185S)-GFP (Fig. 1F). As these mutants could not form alternative intrachain disulphide bonds, it was anticipated that they would be resistant to DTT treatment. Although this increase was much lower than the one observed in Rho(P23H)-GFP, the effect of DTT might reflect a reduction of potential interchain bonds as a result of C185A or C185S substitutions. Overall, these results suggest that both interchain and intrachain disulphide bonds can slow rod opsin mobility in the ER.

### ERdj5 binds to WT rod opsin and dysfunctional ERdj5 affects opsin traffic

**Figure 2.**
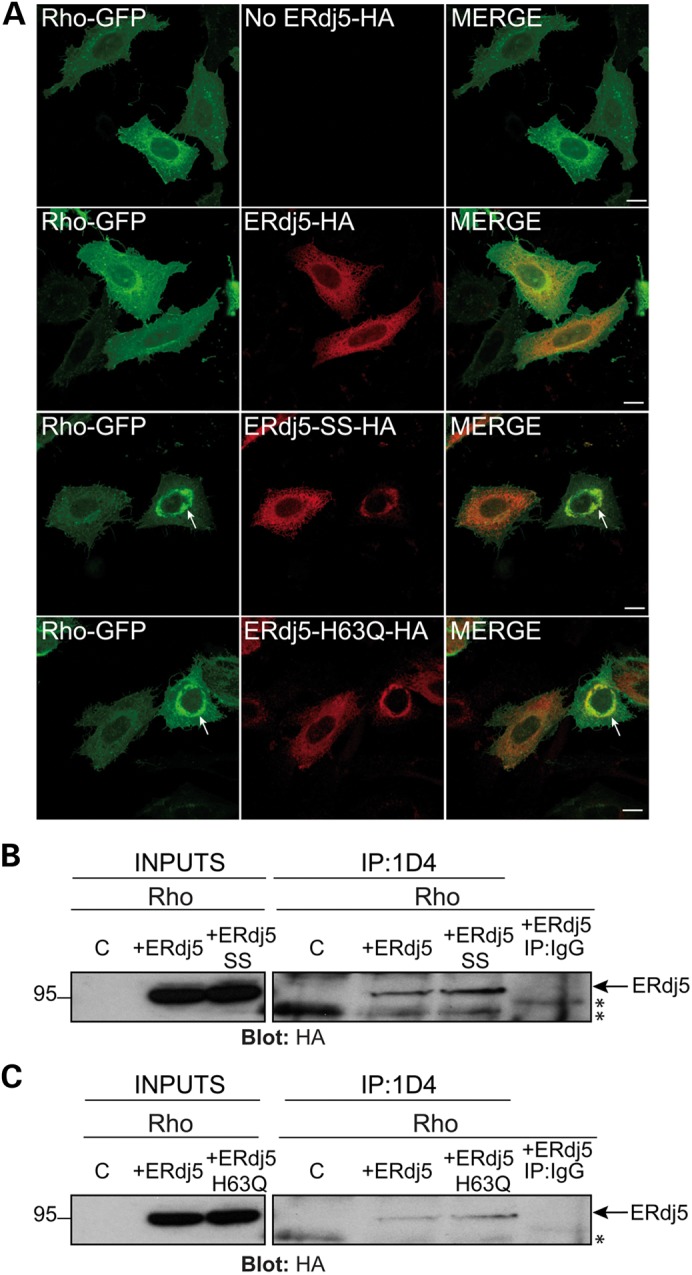
ERdj5 interacts with WT rod opsin and ERdj5 reductase and J domain mutants affect its traffic. (**A**) SK-N-SH cells were transfected with Rho-GFP (green) alone (no ERdj5-HA) or co-transfected at 1 : 1 ratio with ERdj5-HA, ERdj5-SS-HA or ERdj5-H63Q-HA, as indicated. Cells were fixed and immunostained with an antibody against HA for detection of ERdj5 localization (red-middle panel). Arrows highlight ER retention of Rho-GFP. Scale bar: 10 μm. (**B–C**) SK-N-SH cells were transfected with WT rod opsin (Rho) alone (control-C) or co-transfected with Rho and ERdj5-HA or ERdj5-SS-HA or ERdj5-H63Q-HA. Twenty-four hours post-transfection, cell lysates were incubated for 2 h with 1D4 antibody against rod opsin (IP: 1D4) or control IgG (IP: IgG), and immunoprecipitated material was blotted with an antibody against HA for detection of ERdj5. Input loading was 12.5% of IP loading. Arrows highlight the ERdj5 immunoreactive band. Asterisks highlight non-specific immunoreactive bands. The position of molecular weight markers, in kDa, is highlighted on the left.

The effect of C185 substitutions on the trafficking of WT rod opsin and the ER mobility of P23H suggested a requirement for regulation of disulphide bond formation/reduction and subsequently that a reductase protein might be important for rod opsin biogenesis. ERdj5 is an excellent candidate reductase because it links together BiP and EDEM1, two chaperones that have already been shown to affect rod opsin (27,33). To investigate a potential role of ERdj5 in WT rod opsin trafficking, SK-N-SH cells were co-transfected with Rho-GFP alone or co-transfected with Rho-GFP and either ERdj5, the reductase mutant ERdj5-SS or the J domain mutant ERdj5-H63Q (Fig. 2A). All the ERdj5 constructs contained a haemagglutinin (HA) tag at the position before the C-terminal KDEL (37,39,45). When ERdj5 was co-expressed with Rho-GFP, ERdj5 was localized in the ER and did not strongly co-localize with Rho-GFP, which was mainly present at the plasma membrane with only a small amount visible in the ER corresponding to normal synthesis. ERdj5 displayed a reticular pattern, which is characteristic of ER-resident proteins and is consistent with previous studies in HeLa, COS-7 and SH-SY5Y cells (35,37,46). Co-expression of Rho-GFP with the ERdj5-SS, where all cysteine residues in the four CXXC motifs of ERdj5 were substituted to serine (37,45), showed that in some cells Rho-GFP trafficked to the plasma membrane, whereas in others it was retained in the ER and co-localized with ERdj5-SS forming a perinuclear ring-like structure (Fig. 2A). Moreover, the H63Q substitution of the HPD motif within the J domain of ERdj5, which prevents binding to BiP (38,39), also caused ER retention of WT opsin and co-localization with ERdj5-H63Q in the ER (Fig. 2A). Thus, loss of ERdj5's ability to reduce disulphide bonds or to bind to BiP reduced the traffic of WT rod opsin to the plasma membrane by causing its retention in the ER.

The requirement of functional ERdj5 for rod opsin traffic suggested that ERdj5 might form a complex with WT rod opsin. To investigate this hypothesis, SK-N-SH cells were co-transfected with untagged WT rod opsin (Rho) and ERdj5-HA, ERdj5-SS-HA or ERdj5-H63Q-HA. Cell lysates were co-immunoprecipitated with 1D4 antibody against rod opsin and blotted with an antibody for HA (Fig. 2B, C). ERdj5 was co-immunoprecipitated with WT rod opsin; however, the interaction was stronger with the ERdj5-SS and ERdj5-H63Q mutants. No ERdj5 immunoreactivity was detected with the non-specific immunoglobulin G (IgG) pull down (Fig. 2B, C). These results suggest that ERdj5 forms a complex with WT rod opsin and that loss of the reductase activity of ERdj5 or loss of binding to BiP results in a stronger or prolonged association of ERdj5 with WT rod opsin.

### ERdj5 binds to P23H rod opsin and promotes its degradation

**Figure 3.**
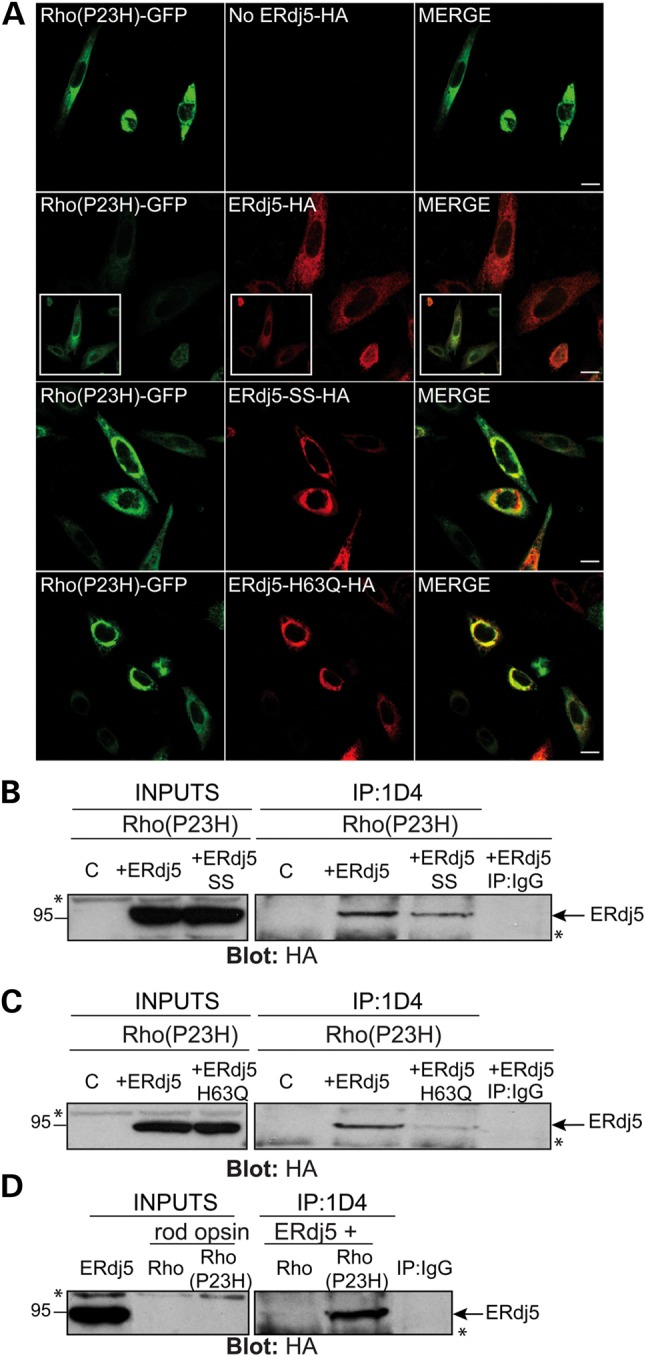
ERdj5 reductase and J domain mutants cause accumulation of P23H rod opsin. (**A**) SK-N-SH cells were transfected with Rho(P23H)-GFP (green) alone (no ERdj5-HA) or co-transfected at 1 : 1 ratio with ERdj5-HA, ERdj5-SS-HA or ERdj5-H63Q-HA and immunostained with an antibody against HA for detection of ERdj5 localization (red-middle panel). Cells were analysed by confocal microscopy keeping the same scanning intensity levels between all conditions. The inset boxes highlight the localization of Rho(P23H)-GFP after increasing the detection sensitivity. Scale bar: 10 μm. (**B–C**) SK-N-SH cells were transfected with Rho(P23H) alone (control-C) or co-transfected with ERdj5-HA, ERdj5-SS-HA or ERdj5-H63Q-HA. (**D**) Comparison of ERdj5 co-immunoprecipitation with Rho or Rho(P23H), as indicated. Twenty-four hours post-transfection, cell lysates were incubated for 2 h with 1D4 antibody against rod opsin (IP: 1D4) or control IgG (IP: IgG), and immunoprecipitated material was blotted with an antibody against HA for detection of ERdj5. Input loading was 12.5% of IP loading. Arrows highlight ERdj5 immunoreactive band. Asterisks highlight non-specific immunoreactive bands. The position of molecular weight markers, in kDa, is highlighted on the left.

ERdj5 can accelerate ERAD of both glycosylated and non-glycosylated misfolded proteins (35,36,38–40). To investigate a possible role of ERdj5 in P23H rod opsin degradation and trafficking, ERdj5 and the mutants ERdj5-SS-HA and ERdj5-H63Q-HA were co-transfected with Rho(P23H)-GFP (Fig. 3A). The image acquisition settings used for immunofluorescence and confocal microscopy were kept the same for all conditions in order to monitor the expression level of Rho(P23H)-GFP and correlate a decrease in fluorescence with a reduction in Rho(P23H)-GFP expression. Overexpression of ERdj5 reduced the fluorescence level of Rho(P23H)-GFP suggesting that P23H degradation might be increased. Imaging at increased intensity levels revealed that the remaining Rho(P23H)-GFP was still retained within the ER (Fig. 3A inset). In contrast, overexpression of ERdj5-SS-HA or ERdj5-H63Q-HA resulted in increased levels of Rho(P23H)-GFP and co-localization with mutant ERdj5 in the ER with enhanced inclusion formation (Fig. 3A). These results suggest a role of ERdj5 in the degradation of P23H rod opsin, which is blocked in the absence of the reductase activity of ERdj5 or when ERdj5 cannot bind BiP. Moreover, ERdj5 was often recruited to Rho(P23H)-GFP inclusions (77% incidence), whereas ERdj5-SS and ERdj5-H63Q always co-localized with Rho(P23H)-GFP in inclusions (100% incidence) (Supplementary Material, Fig. S2).

The presence of ERdj5 in P23H inclusions suggested that ERdj5 might recognize P23H as a misfolded conformer and attempt to promote its degradation, a process that might be blocked by the SS and H63Q mutations and lead to stronger association of P23H with the ERdj5 mutants. To investigate whether ERdj5 forms a complex with P23H, co-immunoprecipitation was performed in cells co-transfected with Rho(P23H) and ERdj5 or Rho(P23H) with the mutants ERdj5-SS and ERdj5-H63Q (Fig. 3B, C). ERdj5-SS and ERdj5-H63Q mutants co-immunoprecipitated with P23H rod opsin but not at higher levels than ERdj5, as expected, and as observed with WT rod opsin (Fig. 2B, C). We believe that this difference is potentially due to insolubility of the complex between P23H and ERdj5 mutants. A similar effect was also observed with an ATPase-mutant of BiP(T37G) and P23H (33). Finally, to address whether ERdj5 binds stronger to WT or P23H rod opsin, co-immunoprecipitation was performed in cells co-expressing WT or P23H with ERdj5, which showed that there was greater recovery of ERdj5 with P23H compared with WT rod opsin, indicating preferential binding to the mutant rod opsin (Fig. 3D).

**Figure 4.**
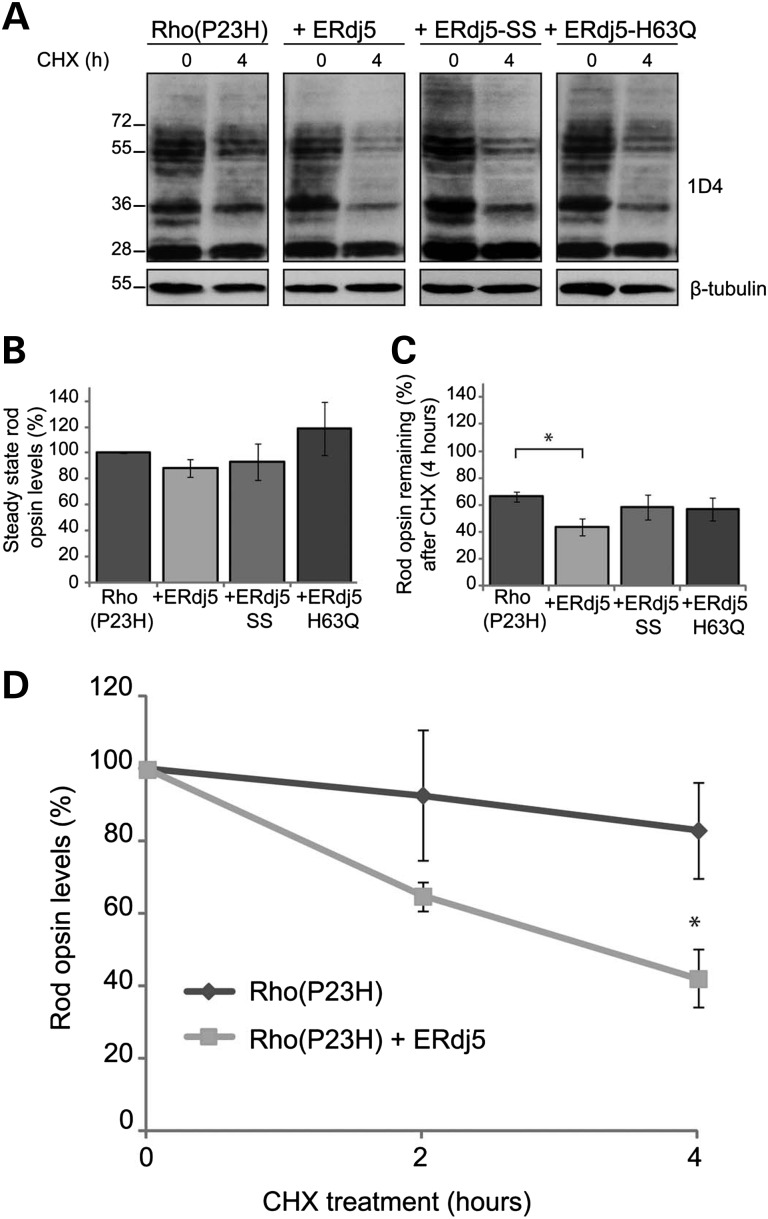
ERdj5 enhances the degradation of P23H rod opsin. SK-N-SH cells were either transfected with untagged P23H rod opsin (Rho(P23H)) or co-transfected at 1 : 1 ratio with Rho(P23H) and ERdj5, ERdj5-SS or ERdj5-H63Q as indicated. Prior to lysis, cells were left untreated or treated with 50 μg/ml CHX for the indicative time (h). Ten micrograms of soluble protein was resolved and probed with the 1D4 antibody against rod opsin. (**A**) Western blot of Rho(P23H) expression following transfection with ERdj5, ERdj5-SS or ERdj5-H63Q and CHX treatment at 0 and 4 h. β-tubulin was used as a loading control. The position of molecular weight markers, in kDa, is highlighted on the left. (**B**) Graphical representation of the steady state levels of Rho(P23H) in the presence of ERdj5, ERdj5-SS or ERdj5-H63Q. The levels are expressed as a percentage of Rho(P23H) at time 0. Error bars represent standard error, *n* > 4. (**C**) Graphical representation of the percentage of Rho(P23H) remaining levels in the presence of ERdj5, ERdj5-SS or ERdj5-H63Q following 4 h treatment with CHX. Error bars represent standard error, *n* > 4.*P < 0.05, student's *t*-test. (**D**) Degradation of Rho(P23H) in the absence or presence of ERdj5 after 0-, 2- and 4 h treatment with CHX. The levels were normalized to 100% at time 0. Error bars represent standard error, *n* > 4.*P < 0.05, student's *t*-test.

To confirm that ERdj5 mediates P23H degradation, cells were co-transfected with untagged P23H rod opsin (Rho(P23H)) and ERdj5, ERdj5-SS or ERdj5-H63Q. Prior to cell lysis, cells were either left untreated or treated for up to 4 h with cycloheximide (CHX), which inhibits protein synthesis and can be used as a tool to investigate differences in half-life and degradation of proteins (Fig. 4). Protein quantification showed that the steady state Rho(P23H) levels were reduced in the presence of ERdj5, whereas they were increased in the presence of ERdj5-SS and ERdj5-H63Q mutants (Fig. 4A, B). Treatment with CHX (4 h) resulted in a 40% reduction of Rho(P23H) levels and a significant enhancement of degradation [60% reduction of Rho(P23H) levels] in the presence of ERdj5 (Fig. 4A, C). Interestingly, the presence of ERdj5-SS or ERdj5-H63Q did not lead to a further reduction of Rho(P23H) levels, as they did not promote Rho(P23H) degradation. When the levels of P23H were normalized to the control (0 h, CHX treatment) to assess the rate of degradation (Fig. 4D), the presence of ERdj5 led to a significant decrease in the amount of Rho(P23H), a 30% drop after 2 h and over 50% after 4 h of CHX treatment (Fig. 4D). Overall, these results suggest that ERdj5 can stimulate the degradation of P23H rod opsin, a process that requires both the reductase activity of ERdj5 and its ability to bind to BiP.

### ERdj5 affects WT and P23H mobility in the ER

**Figure 5.**
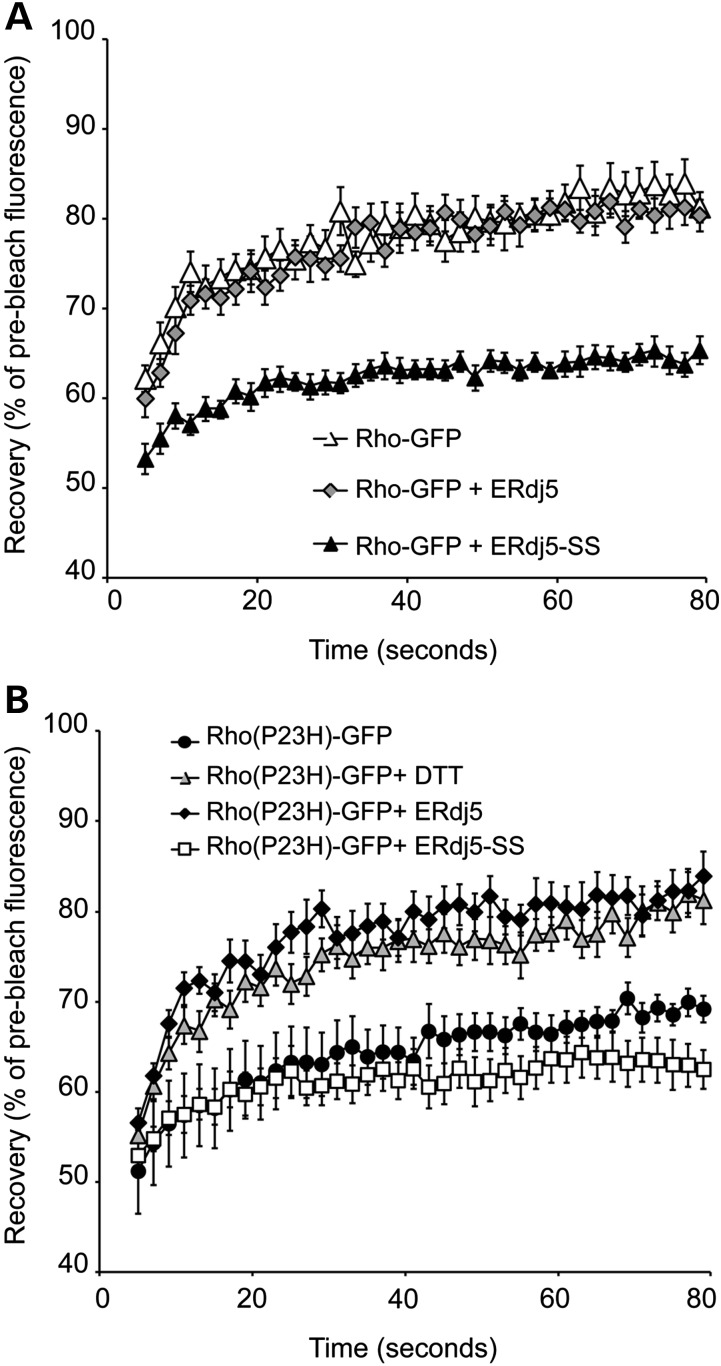
ERdj5 affects rod opsin mobility in the ER. SK-N-SH cells were either transfected with Rho-GFP alone or co-transfected with Rho-GFP and ERdj5 (1 : 2 ratio) or ERdj5-SS (1 : 1 ratio), and 24 h later, live-cell imaging was performed. (**A**) Graphical representation of recovery after photobleach for Rho-GFP (white triangles) and Rho-GFP in the presence of ERdj5 (grey diamonds) or ERdj5-SS (black triangles). Fluorescence intensities for the 2 × 2-μm area were normalized to pre-bleach levels at 100%. Error bars represent standard error, *n* ≥ 12. (**B**) Graphical representation of recovery after photobleach for Rho(P23H)-GFP (black circles), Rho(P23H)-GFP treated with 3 mm DTT (grey triangles) and Rho(P23H)-GFP in the presence of ERdj5 (black diamonds) or ERdj5-SS (white squares). Fluorescence intensities for the 2 × 2-μm area were normalized to pre-bleach levels at 100%. Error bars represent standard error, *n* ≥ 12.

The ER retention of Rho-GFP after co-expression with ERdj5-SS suggested that loss of the reductase activity of ERdj5 might enhance aggregation and reduce Rho-GFP mobility in the ER. In order to investigate this, Rho-GFP was either co-expressed with ERdj5 or ERdj5-SS and FRAP analysis was performed (Fig. 5A). Rho-GFP showed ∼80% recovery of the pre-bleached levels, as previously described (33) and its ER mobility was not altered by ERdj5 overexpression. Conversely, co-expression with the reductase mutant reduced Rho-GFP mobility by 20% as Rho-GFP only recovered 60% of the pre-bleached level (Fig. 5A). This result suggests a requirement for ERdj5 reductase activity to maintain WT rod opsin solubility in the ER.

Moreover, the involvement of ERdj5 in the degradation of P23H rod opsin suggested that this might be accompanied by a reduction in P23H aggregation in the ER. Therefore, FRAP analysis was performed in Rho(P23H)-GFP cells overexpressing ERdj5 or ERdj5-SS (Fig. 5B). The Rho(P23H)-GFP control cells exhibited 60–65% recovery of the pre-bleached levels, which was reduced by 10% after co-expression with the mutant ERdj5-SS. Interestingly, upon ERdj5 overexpression, P23H rod opsin mobility was accelerated and its recovery reached 80% of the pre-bleached values, similar to the recovery rate of Rho-GFP and to that observed in P23H after overexpression of BiP (33). Unlike WT rod opsin, treatment with DTT did not affect the localization or traffic of P23H. Nevertheless, DTT improved P23H mobility with similar efficiency to ERdj5, suggesting that this improvement involves the reductase activity of ERdj5 (Fig. 5B).

To confirm that the above-mentioned alterations in rod opsin mobility were specific to rod opsin and not due to global alteration in the ER membrane fluidity after ERdj5 manipulation or DTT treatment, the same conditions were repeated in cells expressing the ER-associated protein YFP-HSJ1b(274-324) (Supplementary Material, Fig. S3). As previously observed (33), YFP-HSJ1b(274-324) showed a rapid recovery reaching ∼85% of the pre-bleached levels. Its recovery and ER localization remained unaltered after ERdj5 or ERdj5-SS overexpression as well as after incubation with DTT, suggesting that the above-mentioned observations were specific to rod opsin (Supplementary Material, Fig. S3). In conclusion, these results highlight a role of the reductase activity of ERdj5 in improving rod opsin solubility in the ER, preventing its aggregation and promoting its degradation.

### Silencing of ERdj5 leads to accumulation of rod opsin in the ER

**Figure 6.**
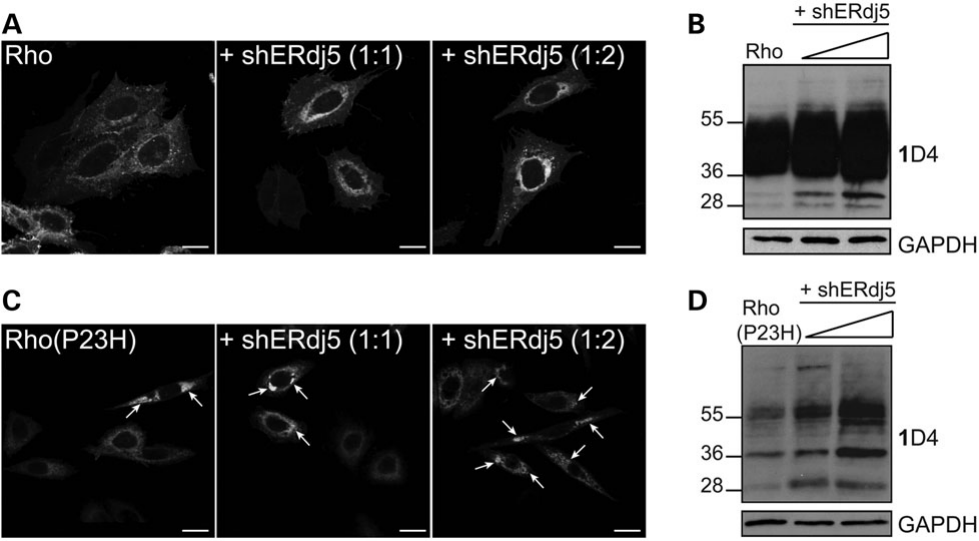
shRNA for ERdj5 results in accumulation of rod opsin. SK-N-SH cells were transfected with rod opsin and empty vector or rod opsin and increasing molar plasmid equivalents (1 : 1, 1 : 2) (arrows) of shERdj5 (+ shERdj5), as indicated. (**A**) Cells expressing Rho-GFP were fixed and analysed by confocal microscopy. Scale bar: 10 μm. (**B**) Cell lysates (10 μg) of Rho in the absence or presence of shERdj5 were resolved and western blotted with 1D4 against rod opsin and GAPDH as a loading control. The position of the molecular weight markers, in kDa, is highlighted on the left. (**C**) Cells expressing Rho(P23H)-GFP were fixed and analysed by confocal microscopy. Arrows highlight Rho(P23H)-GFP inclusions. Scale bar: 10 μm. (**D**) Cell lysates (10 μg) of Rho(P23H) in the absence or presence of shERdj5 were resolved and blotted with 1D4 against rod opsin and GAPDH as a loading control. The position of the molecular weight markers, in kDa, is highlighted on the left.

To investigate the effect of endogenous ERdj5 in rod opsin biogenesis, a short hairpin RNA (shRNA) was produced and used to silence the expression of human ERdj5 via RNA interference. Initially, the shERdj5 was tested in SK-N-SH cells and found to be effective in reducing ERdj5 RNA and protein levels up to 90% (Supplementary Material, Fig. S4). Co-transfection of Rho-GFP and increasing amounts of shERdj5 led to retention of Rho-GFP in the ER (Fig. 6A). Reduction of ERdj5 led to a global increase of WT Rho levels and especially the ∼36-kDa species, which corresponds to the EndoH-sensitive glycoform of WT rod opsin that has not trafficked beyond the ER (Fig. 6B) (33). Additionally, silencing of ERdj5 led to increased levels of Rho(P23H)-GFP in the ER and an increase in the number of Rho(P23H)-GFP inclusions compared with Rho(P23H)-GFP control cells (Fig. 6C), as well as a global increase of the ∼28-, ∼36- and ∼45–60-kDa species of Rho(P23H), which correspond to an N-terminal truncated protein, the EndoH-sensitive glycoform and dimer, respectively (Fig. 6D) (17,29,47). These findings suggest that silencing of ERdj5 can affect the traffic of WT rod opsin and can increase accumulation of P23H in the ER, thus making it more prone to aggregation.

## DISCUSSION

The ER is a compartment that is critical for protein folding, assembly and disulphide bond formation. However, a fraction of newly synthesized proteins never reach the correct native state and are subjected to retrotranslocation from the ER and ERAD (48). Efficient retrotranslocation of misfolded proteins requires reduction of disulphide bonds and protection of reduced cysteine residues from further reaction prior to dislocation. The formation of other unwanted intrachain or interchain disulphide bonds must also be avoided (16). Such a requirement is likely to be important for retrotranslocation of misfolded rod opsin, as it is characterized by an non-native disulphide bond between C185 and C187 as opposed to the native disulphide bond between C110 and C187 (1,2). The data presented here suggest that the co-chaperone and reductase ERdj5 promotes the degradation and prevents the aggregation of P23H rod opsin and is also involved in the processing of WT rod opsin.

ERdj5 is an important component of the ER quality control machinery. It is the largest of the PDI family of proteins and the only one to contain a J domain and multiple active thioredoxin domains within the same structure (35–38). These unique structural characteristics allow ERdj5 to catalyse the reduction of disulphide bonds of misfolded non-glycoproteins or glycoproteins received from EDEM1 and transfer the reduced proteins to BiP in an ATP-dependent manner (36,40). Both functions seem to play a role in the normal processing of WT rod opsin as co-expression with ERdj5-SS and ERdj5-H63Q mutants that block its reductase and its co-chaperone activity, respectively, resulted in retention and accumulation of a fraction of WT rod opsin in the ER. The same effect on WT rod opsin was observed after shERdj5-mediated silencing of ERdj5. Additionally, while overexpression of the wild-type form of ERdj5 did not promote P23H folding as seen with EDEM1 (27), it accelerated P23H degradation. P23H degradation was blocked by ERdj5 mutants, and ERdj5 knockdown led to a further accumulation of P23H in the ER. This is consistent with another study, which showed that ERdj4 and ERdj5 accelerated the degradation of the non-glycosylated misfolded surfactant protein C (SP-C), and the effect was reversed in the presence of ERdj4-H54Q and ERdj5-H63Q mutants or by downregulation of ERdj4 and ERdj5 (39). Another study reported that overexpression of ERdj5 accelerated the degradation of two glycoproteins, the null Hong Kong (NHK) variant of human α-1 antitrypsin and the J chain of mouse immunoglobulin M (38). For both these client proteins, degradation was blocked in the presence of ERdj5-SS and ERdj5-H63Q mutants suggesting that both functions are required, because ERdj5-H63Q that retains the reductase activity failed to accelerate ERAD (38).

In the present study, these ERdj5 mutants had the same effect on blocking the degradation of P23H, implying that both functional domains are required for P23H ERAD. Moreover, further support for the role of ERdj5 in P23H degradation is evident from co-localization of ERdj5 and ERdj5 mutants in P23H inclusions. Previously, it has been shown that other molecular chaperones can be recruited to P23H inclusions such as Hsp70, HSJ1a and HSJ1b (17,49). The presence of ERdj5 in P23H inclusions as well as the effect of ERdj5 downregulation or functional inhibition suggested that ERdj5 might form a complex with rod opsin. Co-immunoprecipitation studies showed that ERdj5 binds to WT rod opsin suggesting that ERdj5 has a role in normal rod opsin biogenesis. This binding was not dependent on the presence of active CXXC motifs or a functional J domain as ERdj5-SS and ERdj5-H63Q mutants also co-immunoprecipitated with WT rod opsin. The stronger association of WT rod opsin with the ERdj5-SS mutant could indicate prolonged binding owing to its lack of ability to act as a reductase. Additionally, prolonged binding of WT rod opsin to the ERdj5-H63Q could suggest lack of ability to facilitate substrate release without BiP binding and potential client protein handover. These results for WT rod opsin are consistent with the ER retention observed upon co-expression of the ERdj5 mutants with the LDLR (41). Moreover, ERdj5 bound preferentially to mutant P23H rod opsin; however, this binding was not enhanced by the ERdj5-SS and ERdj5-H63Q mutations. This was unexpected, as it is anticipated that ERdj5 would bind P23H to promote ERAD and release would require reduction of disulphide bonds and BiP binding. We believe that the failure to detect stronger binding might be due to the insolubility of these complexes, as the same effect was observed with an ATPase-mutant of BiP (33).

Further support for this hypothesis is provided by the reduced mobility of Rho(P23H)-GFP in the ER after co-expression with the reductase mutant. Importantly, the converse effect on P23H mobility was observed after ERdj5 overexpression, by prevention of the non-native C185–C187 disulphide bond or after short incubation with the reducing agent DTT. A similar effect for DTT was reported with the misfolded vesicular stomatitis virus G (VSVG) (44,50). DTT had some effect on Rho(P23H;C185A)-GFP and Rho(P23H;C185S)-GFP mobility, despite these mutants not being able to form alternative intrachain disulphide bonds. Previously, DTT treatment has been shown to accelerate the ER mobility of Galt-GFP, which does not contain any intrachain disulphide bonds, after incubation with brefeldin A (BFA), which blocks exit of proteins in the ER (50). The improvement of Rho(P23H;C185A)-GFP and Rho(P23H;C185S)-GFP mobility after DTT treatment might reflect a general change in the ER membrane viscosity after reduction of disulphide bonds. However, DTT treatment did not affect the mobility of the ER-associated protein HSJ1b, suggesting no gross changes in membrane fluidity. Therefore, DTT might have a more specific effect on proteins that are no longer associated in dynamic crosslinked aggregates or proteins that form incorrect interchain bonds (50,51). These findings suggest that non-native inter- and intra-chain disulphide bonds are a factor for P23H immobilization in the ER and that the reductase activity of ERdj5 is important in maintaining P23H solubility in the ER.

Interestingly, while overexpression of ERdj5 had no effect on Rho-GFP mobility in the ER, co-expression with ERdj5-SS reduced WT mobility implying a requirement of a fraction of WT rod opsin for disulphide bond reduction for efficient trafficking. Previous studies have shown that C185A and C185S mutations were able to fold efficiently, bind to 11-*cis* retinal and regenerate rhodopsin (1,3). The C185A mutant, however, which was more extensively characterized, was found to be less thermally stable than WT rod opsin with slow affinity rates for 11-*cis* retinal. The C185A mutation appeared to destabilize the open-pocket conformation of opsin leading to an equilibrium between correctly folded and denatured states of opsin (1,3). Therefore, it is possible that the correct disulphide bond between C110 and C187 residues is formed only in a fraction of Rho-GFP, which traffics efficiently and quickly to the plasma membrane, whereas the fraction that forms the C185–C187 or C110–C185 bond belongs to off-pathway folding intermediates that limit folding to the native state in the ER. This might explain the live-cell imaging results in the present study, which showed faster trafficking of Rho(C185A)-GFP and Rho(C185S)-GFP mutants to the plasma membrane, as opposed to Rho-GFP, and similar to the effect of reducing conditions with DTT on Rho-GFP. Additionally, the presence of the off-pathway folding intermediates in the ER might also explain why the Rho-GFP mobile fraction does not fully recover and its levels only reach ∼80% of the pre-bleached values.

Therefore, it is likely that ERdj5 prevents WT rod opsin from forming incorrect intra- or inter-molecular bonds while it is in the ER. Its association with rod opsin folding intermediates and its association with BiP could help rod opsin to maintain its solubility in the ER until further chaperones and folding factors complete the folding of rod opsin (33). The interaction of ERdj5 with P23H rod opsin also supports the hypothesis that ERdj5, like ERdj3 and ERdj4, can bind directly to misfolded protein substrates, thus acting as a chaperone and co-chaperone that recruits BiP (39,52,53). Previously, it has been proposed that ERdj5-mediated ERAD requires EDEM1 to function because inhibition of mannose trimming by kifunensine blocked ERAD of NHK and immunoglobulin J chains even when ERdj5 was overexpressed (38). However, it has been shown that EDEM1 recognition of misfolded substrates does not require mannose trimming, but instead, this can be mediated by exposed hydrophobic domains (54). Two lines of evidence support this hypothesis. Both mannose trimming and glycan chains were not required for P23H rod opsin degradation or for P23H-EDEM1 binding (27). More recently, it has been shown that the degradation of glycoprotein substrates such as NHK can be mediated via the BiP/ERdj5 pathway, which serves as a back-up ERAD pathway upon ER stress (40).

**Figure 7.**
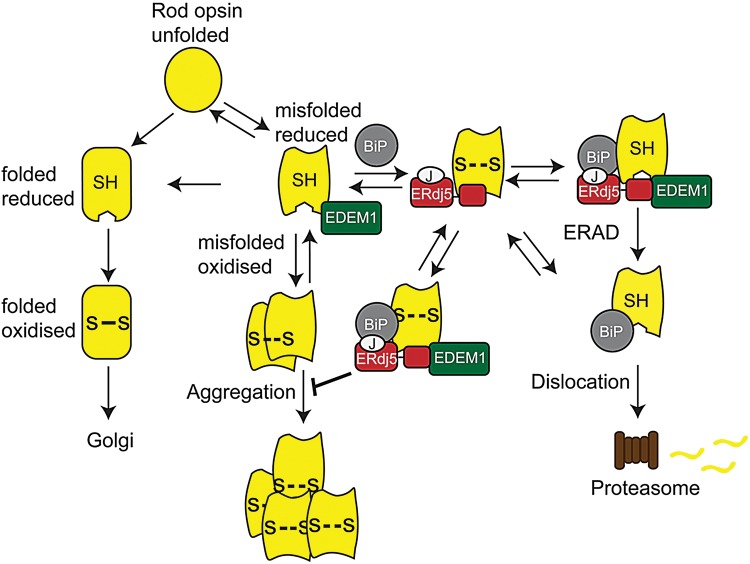
Model of molecular chaperone modulation on rod opsin biogenesis. Left side: rod opsin could fold correctly, acquire the correct disulphide bond (C110–C187; **S-S**) and translocate through the secretory pathway. Right side: alternatively, it might misfold and form either an incorrect intrachain (C185–C187 or C110–C185**; S- -S**) or interchain disulphide bonds (**S- -S)**. ERdj5 reduces the aberrant disulphide bonds **(SH)**. Rod opsin can either refold to its correct conformation or be subjected to ERAD via the ERdj5/BiP or EDEM1/ERdj5/BiP pathway. EDEM1 binds to the C-terminus of ERdj5, whereas BiP binds to the J domain. Upon BiP ATP hydrolysis, rod opsin is dislocated and degraded by the proteasome. Alternatively, EDEM1 may bind misfolded rod opsin directly and correct the folding or promote the degradation of rod opsin. If molecular chaperones are not recruited, misfolded rod opsin could be retained in the ER leading to rod opsin aggregation.

Therefore, the data presented here in combination with previous findings on the role of EDEM1 and BiP on rod opsin (27,33) suggest the formation of a proteostasis network between BiP, ERdj5 and EDEM1 that is important for rod opsin biogenesis and quality control. A previously described model of this network's potential roles in the quality control of misfolded proteins (36) has been modified for rod opsin as the client protein and is illustrated in Figure 7. Rod opsin could fold correctly, acquire the correct disulphide bond (C110–C187) and traffic through the secretory pathway to the Golgi and to the plasma membrane with little or no chaperone assistance. Alternatively, rod opsin could misfold and amino acid substitutions, such as class II mutations, would increase the potential risk of this occurring. The misfolded rod opsin could form either incorrect intrachain (C185–C187 or C110–C185) or interchain disulphide bonds. In that case, ERdj5 reduces the aberrant disulphide bonds and rod opsin could either refold to its correct conformation or be subjected to ERAD via the EDEM1-ERdj5-BiP pathway or the ERdj5-BiP pathway. Alternatively, EDEM1 may bind misfolded rod opsin directly and correct the folding or promote the degradation of rod opsin. If molecular chaperone-mediated quality control fails, misfolded rod opsin could accumulate in the ER leading to rod opsin aggregation in the ER or in the cytoplasm following retrotranslocation and failed proteasomal degradation.

Collectively, the data presented here identify ERdj5 as the third member of a specialized proteostasis network (BiP-ERdj5-EDEM1) that regulates rod opsin biogenesis, facilitates rod opsin quality control and reduces mutant rod opsin aggregation. Manipulation of this network might be a viable therapy for these currently untreatable forms of blindness.

## MATERIALS AND METHODS

### Materials

The mouse monoclonal primary antibody 1D4 (1 : 1000) against rod opsin was a gift from Professor Robert Molday (Department of Biochemistry and Molecular Biology, University of British Columbia, Canada). Cycloheximide, DTT, protease inhibitor cocktail (PIC) and antibodies to HA (H6908) (1 : 1000), to GFP (G1546) (1 : 1000), to β-tubulin (T8328) (1 : 2000) and to GAPDH (G8735) (1 : 40 000) were from Sigma (Poole, UK). Lipofectamine, Plus reagent, goat anti-mouse Alexa Fluor 594 secondary antibody-conjugated IgG (1 : 1000) and protein G magnetic Dynabeads were purchased from Invitrogen (Paisley, UK). Endoglycosydase (EndoH) and Peptide N-glycosidase F (PNGase F) were from New England Biolabs (Herts, UK). The bicinchonitic acid (BCA) protein assay kit and goat anti-mouse secondary antibody conjugated to horseradish were from Pierce (Thermo Scientific Cramlington, UK). The Quikchange II site-directed mutagenesis kit was from Agilent Technologies (Berkshire, UK). The RNeasy Mini Kit and QuantiTect Reverse Transcription Kit were from Qiagen (Manchester, UK). The ECL Plus reagent kit was from GE Healthcare (Buckinghamshire, UK).

### Expression construct, site-directed mutagenesis and shRNA production

Rod opsin constructs, untagged rod opsin in pMT3 (Rho) and rod opsin-GFP (Rho-GFP), were as described previously (17). The Rho(C185A)-GFP, Rho(C185S)-GFP, Rho(P23H;C185A)-GFP, Rho(P23H;C185S)-GFP expression plasmids were generated by site-directed mutagenesis using either the Rho-GFP or the Rho(P23H)-GFP construct as template. All constructs were confirmed by sequencing. The mERdj5-HA and mERdj5-SS expression plasmids were as described (39). The hERdj5-HA and hERdj5-H63Q expression plasmids were a gift from Dr Weaver (CCMC, Ohio). YFP-HSJ1b(274-324) was as described previously (33). For shERdj5 production, two sets of oligos (forward and reverse) were designed based on the GGAGGAGAUUGUUUGACUU published siRNA sequence (55). The sequence of the forward oligo included the siRNA sequence in sense (5′) and antisense (3′) orientation separated by the CTCGAG nucleotide-specific loop sequence (hairpin). The oligos were ordered from MWG Eurofins (Abingdon, UK) and were cloned to pSUPER vector (OligoEngine, Washington). The presence of the correct insert within the recombinant pSUPER vector was confirmed by sequencing using the M13 primer GTAAAACGACGGCCAGT.

### Cell culture, transfection and immunofluorescence

SK-N-SH human neuroblastoma cells were maintained, transfected and imaged essentially as previously described (27). Briefly, cells were seeded on chamber slides at a density of 50 000 cells/well and transfected with 200 ng of total DNA. Twenty-four hours after transfection, cells were fixed with ice-cold methanol or 4% paraformaldehyde and permeabilized with Triton X-100 (0.5%) (Sigma, UK) in phosphate-buffered saline (PBS) (137 mm sodium chloride, 2.7 mm potassium chloride, 8.1 mm di-sodium hydrogen phosphate, 1.5 mm potassium dihydrogen phosphate; OXOID, UK). Non-specific binding was blocked for 1 h with PBS containing 3% (w/v) bovine serum albumin (BSA) and 10% (v/v) normal donkey serum. Anti-HA antibody (1 : 1000) primary antibody in blocking buffer was added for 1 h followed by washing with PBS, and secondary antibody Alexa Fluor 594-conjugated IgG was used at 1 : 1000 in blocking buffer for 1 h. Cells were washed twice with PBS and once with DAPI at a concentration of 2 μg/ml in PBS prior to mounting in fluorescent mounting medium (DAKO, Cambridgeshire, UK). Fluorescence was observed on a Carl Zeiss LSM 700 confocal laser-scanning microscope for image acquisition. Images were exported from LSM browser to Adobe Photoshop for figure preparation and annotation in Adobe Illustrator.

### FRAP analysis

SK-N-SH cells were seeded on MatTek (MatTex Corp, MA, USA) slide dishes at a density of 600 000 cells/dish and after 24 h were transfected with 2000 ng of total DNA. Live imaging and FRAP was performed 24 h after transfection as previously described (33). For the DTT treatment, 3 mm DTT was administered 15 min prior to live imaging.

### SDS–PAGE and western blot

Cells were lysed with 1% (w/v) n-Dodecyl-β-D-maltoside (DM) lysis buffer containing 2% (v/v) mammalian PIC, 2% (v/v) mammalian phosphatase inhibitor cocktail and 5 mm ethylenediamine tetraacetic acid (EDTA) in PBS. Cell lysates were centrifuged for 15 min at 12 000×*g* and at 4°C. Protein concentration was measured with the BCA Protein Assay kit (Pierce) following the manufacturer's instructions. For the deglycosylation reactions, 10 μg of total protein in DM soluble cell lysate was digested with PNGaseF or EndoH for 2 h at 37°C. Cell lysates were diluted in 2× sodium dodecyl sulphate (SDS) sample loading (10% SDS, 20% glycerol, 0.1% (w/v) bromophenol blue, 0.5 m Tris–HCl, pH 6.8, 2.5% (v/v) 2-β mercaptoethanol), to a final concentration of 1×. Samples for rod opsin blot were not denatured, whereas samples for all the other antibodies were heated at 95°C for 5 min before they resolved by SDS–polyacrylamide gel electrophoresis (SDS–PAGE) and western blot. Proteins were transferred to nitrocellulose with 25 mm Tris, 192 mm glycine, 0.01% SDS (w/v) and 20% (v/v) methanol. To prevent non-specific binding, membranes were blocked at 4°C with 5% (w/v) Marvel Milk in PBS with 0.1% (v/v) tween and immunodetection with the proteins of interest was carried out using the primary and secondary antibodies described in Materials section. Proteins were detected using ECL Plus reagent (GE Healthcare, UK). For the CHX assay, cells were treated with 50 μg/ml CHX (Sigma, UK) for 2 and 4 h prior to lysis. Each experiment was performed at least four times. Protein quantitation was carried out using ImageJ.

### Immunoprecipitation

Protein G magnetic Dynabeads (Invitrogen, UK) were blocked overnight with 0.2% (w/v) BSA and 0.02% (v/v) tween 20 in PBS at 4°C in an end-over-end rotor. Cells were lysed with 1% (w/v) DM lysis buffer containing 5% (v/v) mammalian PIC and 5 mm EDTA in PBS. After lysis, some lysate was kept for the input fraction and after the addition of 2× SDS buffer was stored in −20°C. The remaining lysate was separated in different IP conditions and incubated for 2 h at room temperature or overnight at 4°C in an end-over-end with the pre-blocked Dynabeads and with 1–4 μg of specific antibody or 1–4 μg of non-specific IgG. Following incubation, the bead–antibody–protein complexes were washed four times with 1% DM washing buffer. Samples were eluted from the beads with 30 μl of 2× SDS buffer, vortexed for 5 s and spun for 1 min at 12 000×*g*. Supernatant was collected via the Magnarak to minimize magnetic bead contamination. Samples for rod opsin blot were not denatured, whereas samples for all the other antibodies were heated at 95°C for 5 min prior to SDS–PAGE and western blot.

### Quantitative reverse transcriptase PCR

SK-N-SH cells were seeded at a density of 100 000 cells per well and transfected with 600 ng/well total DNA prior to cell lysis and RNA extraction. The RNeasy Mini Kit (Qiagen, UK) was used for RNA extraction of cell pellets (10^5^ cells), and cDNA preparation was performed using QuantiTect Reverse Transcription Kit (Qiagen). Relative quantification by real-time PCR of ERdj5 against endogenous β-actin-expression levels was measured using the following sets of primers: human ERdj5 forward (5′ TGAACTACTTTCGGCAAAGAGA 3′) and reverse (5′ TTTCAGCTCAGGATCATTTGAA 3′); human beta-actin forward (5′ CCAACCGCGAGAAGATGA 3′) and reverse (5′ CCAGAGGCGTACAGGGATAG 3′). The relative expression between comparable samples in relation to the expression of the reference gene and gene of interest was calculated through the formula: 2^−ΔΔCt^.

## SUPPLEMENTARY MATERIAL

Supplementary Material is available at *HMG* online.

## FUNDING

This work was supported by Fight for Sight, RP Fighting Blindness, The Big Lottery Fund and The Wellcome Trust (to M.E.C.). Funding to pay the Open Access publication charges for this article was provided by UCL Library.

## Supplementary Material

Supplementary Data
