# Supplementary material for: The co-chaperone and reductase ERdj5 facilitates rod opsin biogenesis and quality control
Source: Hum Mol Genet. 2014 Jul 23;23(24):6594–606. doi: 10.1093/hmg/ddu385 (PMC4240209; doi:10.1093/hmg/ddu385)
Supplement: Supplementary Data [file supp_23_24_6594__index.html]

The co-chaperone and reductase ERdj5 facilitates rod opsin biogenesis and quality control — The co-chaperone and reductase ERdj5 facilitates rod opsin biogenesis and quality control — Supplementary Data 

# The co-chaperone and reductase ERdj5 facilitates rod opsin biogenesis and quality control

## Supplementary Data

Supplementary Data

**Files in this Data Supplement:**

- Supplementary Data - Pdf file
