# Supplementary material for: The co-chaperone and reductase ERdj5 facilitates rod opsin biogenesis and quality control
Source: Hum Mol Genet. 2014 Jul 23;23(24):6594–606. doi: 10.1093/hmg/ddu385 (PMC4240209; doi:10.1093/hmg/ddu385)
Supplement: Supplementary Data [file supp_ddu385_ddu385supp.pdf]

Supplementary Material

The co-chaperone and reductase ERdj5 facilitates rod opsin biogenesis and quality control

Dimitra Athanasiou, Dalila Bevilacqua, Monica Aguila, Caroline McCulley, Naheed Kanuga, Takao Iwawaki, J. Paul Chapple, and Michael E. Cheetham

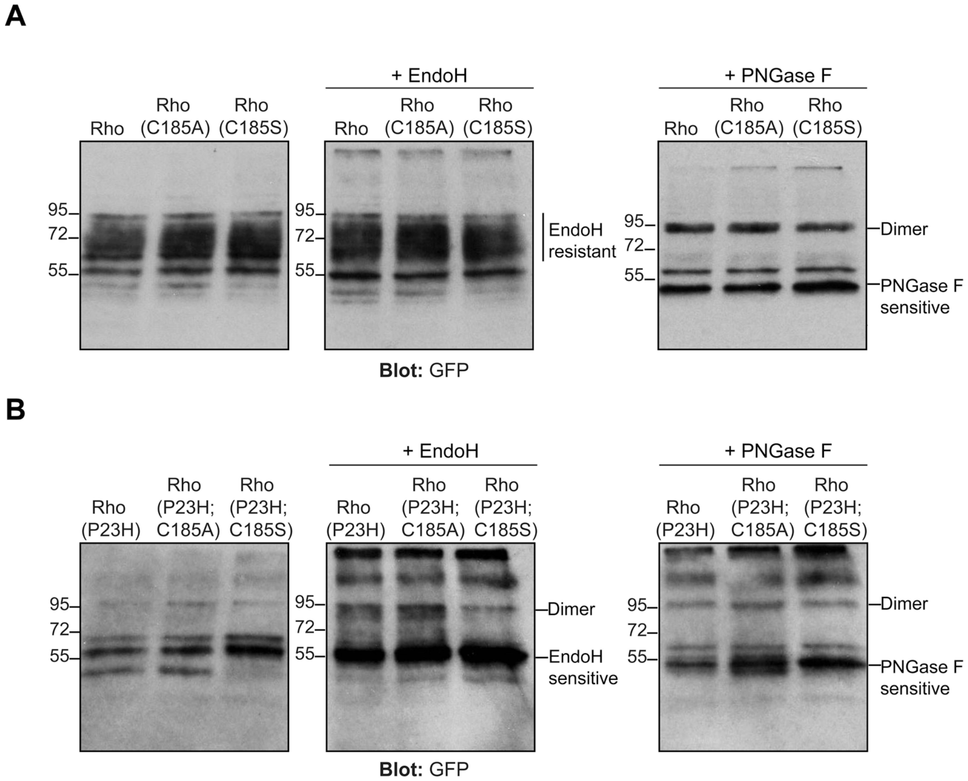

Figure S1. ***C185A and C185S mutations do not affect the glycosylation of rod opsin.***

SK-N-SH cells were transfected with either (**A**) Rho-GFP, Rho(C185A)-GFP and Rho(C185S)-GFP or (**B**) Rho(P23H)-GFP, Rho(P23H;C185A)-GFP and Rho(P23H;C185S)-GFP. 24 hours after transfection cells were lysed and digested for 2 hours at 37ºC with either PNGase F or with EndoH in order to determine empirically the different glycoforms of rod opsin. Undigested, digested PNGase F (+ PNGase F) and EndoH (+ EndoH) lysates (10 µg) were resolved and blotted with an antibody against GFP. The position of the molecular weight markers, in kDa, is shown on the left.

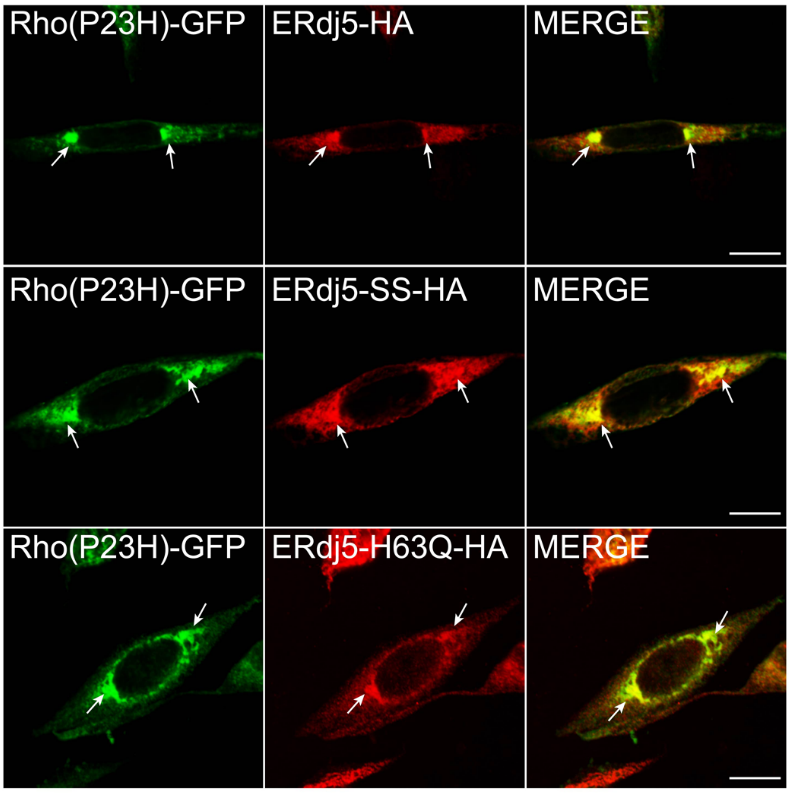

Figure S2. ***Recruitment of ERdj5 and ERdj5 mutants to P23H rod opsin inclusions***

SK-N-SH cells were co-transfected with Rho(P23H)-GFP (green) and either ERdj5-HA, ERdj5-SS-HA or ERdj5-H63Q-HA (all at 1:1 ratio) and immunostained with an antibody against HA to detect ERdj5 (red). Arrows highlight Rho(P23H)-GFP inclusions that are ERdj5 positive. The incidence of Rho(P23H)-GFP and ERdj5, ERdj5-SS-HA and ERdj5-H63Q-HA co-localisation in 100 cells containing inclusions was 77%, 100% and 100% respectively. Cells were analysed by confocal microscopy. Scale bar 10 μm.

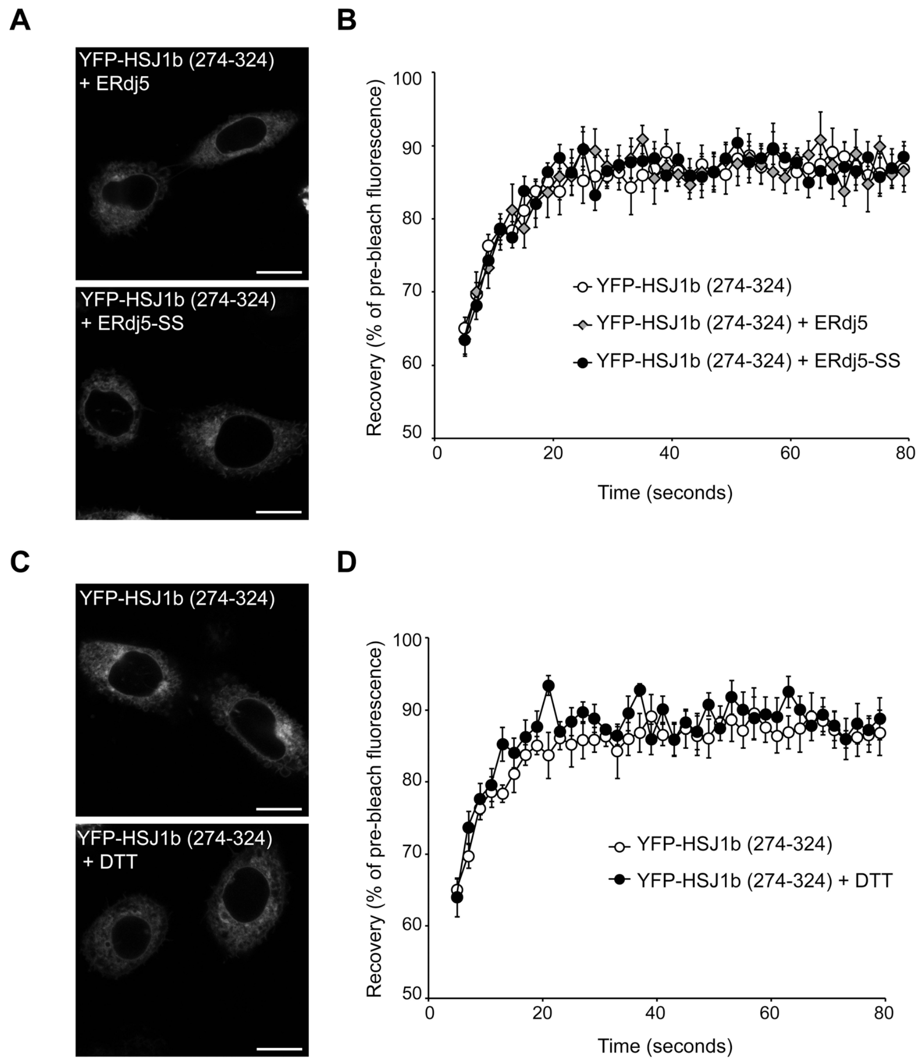

Figure S3. ***ERdj5 manipulation does not affect the localisation and mobility of another ER-associated protein***

(**A**) Representative live cell images of localisation YFP-HSJ1b(274-324). This was not affected by ERdj5 or ERdj5-SS overexpression. Scale bar 10 µm. (**B**) Graphical representation of recovery after photobleach for YFP-HSJ1b(274-324) (white circles) compared to YFP-HSJ1b(274-324) co-transfected with ERdj5 (grey diamonds) or ERdj5-SS (black circles). Fluorescence intensities for the 2x2 µm area of the ER were normalised to pre-bleach levels at 100%. Error bars represent standard error, n≥12. (**C**) Representative live cell images of YFP-HSJ1b(274-324) localisation. This was not affected by DTT treatment. Scale bar 10 µm. (**D**) Graphical representation of recovery after photobleach for YFP-HSJ1b(274-324) (white circles) compared to YFP-HSJ1b after 15 minutes treatment with 3 mM DTT (black circles). Fluorescence intensities for the 2x2 µm area of the ER were normalised to pre-bleach levels at 100%. Error bars represent standard error, *n*≥12.

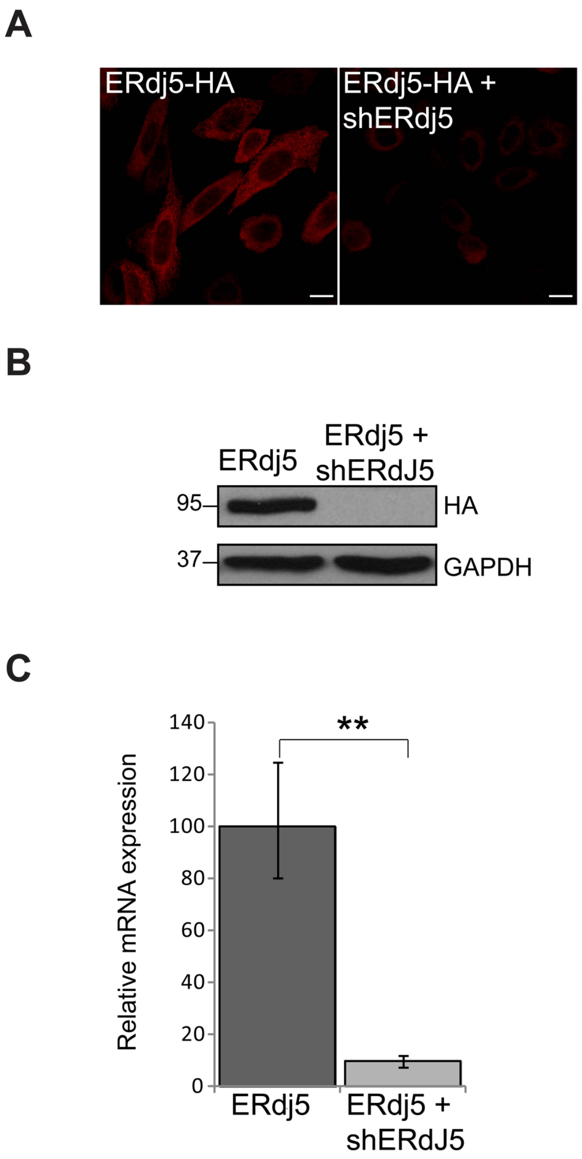

Figure S4. ***shERdj5 reduces the expression of ERdj5***

SK-N-SH cells were transfected with ERdj5-HA or ERdj5-HA and shERdj5 as indicated. (**A**) Cells were stained with the HA antibody for detection of ERdj5 (red) and analysed by confocal microscopy keeping the same scanning settings between all the conditions. Scale bar 10 µm. (**B**) Cell lysates were blotted with HA antibody for detection of ERdj5 expression and with GAPDH as a loading control. The position of molecular weight markers, in kDa, is highlighted on the left. (**C**) Relative mRNA expression of ERdj5 in SK-N-SH cells after transfection with ERdj5-HA or ERdj5-HA and shERdj5, as indicated. Error bars represent standard error, n =3, **p<0.01, Student's *t*-test.
